# Pectin-rich purple passion fruit mesocarp/agarose: film properties and impact of coatings on banana preservation

**DOI:** 10.1039/d5ra09755j

**Published:** 2026-04-23

**Authors:** Bao-Tran Pham-Tran, Nhu-Quynh Thi Nguyen, Nhu-Ngoc Quynh Nguyen, Long Giang Bach, Thuong Thi Nguyen

**Affiliations:** a Nguyen Tat Thanh University Center for Hi-Tech Development Saigon Hi-Tech Park Ho Chi Minh City Vietnam; b Institute of Applied Technology and Sustainable Development, Nguyen Tat Thanh University Ho Chi Minh City 71516 Vietnam blgiang@ntt.edu.vn; c Faculty of Chemical Engineering and Food Technology, Nong Lam University Ho Chi Minh City 70000 Vietnam; d Faculty of Applied Science and Technology, Nguyen Tat Thanh University Ho Chi Minh City 71514 Vietnam nthithuong@ntt.edu.vn

## Abstract

This work aimed to extract pectin from purple passion fruit mesocarp (PPMP) and then blend it with agarose to obtain an eco-friendly material with desirable properties suitable for banana preservation. The PPMP exhibits a high methyl esterification degree (66.79%) and yield (29.83%) under optimal conditions consisting of a dry peel powder particle size of 0.25 mm, a mesocarp powder-to-citric acid solution ratio of 1 : 25 (g mL^−1^), extraction at 90 °C for 60 min, followed by precipitation with 96% ethanol. To overcome the hydrophilicity and limited mechanical strength of neat PPMP films, agarose (A) is blended at varying concentrations of 0.5%, 1%, and 1.5% (w/v). Among the tested formulations, PPMP-A1.5 exhibited the best overall performance, exhibiting increased tensile strength (from 1.15 MPa for neat PPMP to 1.71 MPa), enhanced oxygen-barrier performance (OP reduced from 32.93 × 10^−12^ to 21.60 × 10^−12^ mol m^−1^ s^−1^ Pa^−1^), and improved surface hydrophobicity (contact angle increased from 26.1° to 77.2°), despite certain trade-offs in flexibility and water vapor permeability. Preservation tests further confirm that the PPMP-A1.5 coating effectively prolonged the shelf life of bananas by up to 8 days at 25 °C, minimizing changes in peel browning, firmness, ripening rate, and pH. Notably, the weight loss of bananas coated with PPMP-A1.5 on day 6 is 11.69%, which is significantly lower than that of the uncoated samples (18.04%). These findings underscore the potential of PPMP/agarose coatings as safe, biodegradable, and sustainable packaging materials, serving as an alternative to conventional plastics and aligning with the principles of the circular economy.

## Abstract 2

This study is the first to optimize agarose concentration in pectin-rich matrices derived from purple passion fruit mesocarp for functional coating films and to apply them in prolonging the shelf life of bananas.

## Graphical abstract

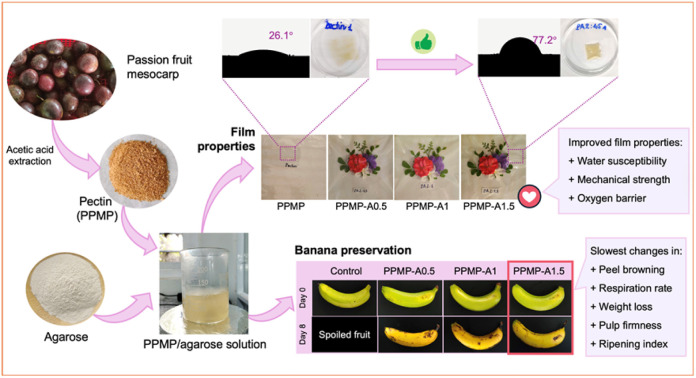

## Introduction

In the past few decades, energy production and packaging manufacturing have been inevitably linked to petroleum resources, the availability of which is steadily declining. Without renewable alternatives, many industries will struggle to meet the increased demand for vital products in the future. Additionally, the use of petrol-based plastics can cause the global problem of environmental pollution due to their slow degradation.^1^ Therefore, identifying and utilizing what is currently described as waste or under-utilized, which could reduce petroleum-dependent effects, is crucial. To date, current research on packaging materials is shifting towards the exploration of next-generation biomaterials, which utilize existing and sustainable waste streams as a source of renewable polymers.

In Vietnam, passion fruit cultivation is dominated by the purple variety (*Passiflora edulis* f. *edulis*), with an annual production of approximately 188 900 tons (according to Vietnam Agriculture, 2024 (ref. 2)). More than 80% of passion fruit in Vietnam is consumed domestically, primarily processed into juice, passion fruit powder, and dietary supplements.^3^ This process generates a considerable amount of waste, primarily peels, which account for 50–60% of the total fruit weight.^4^ Beyond causing environmental pollution when disposed of in landfills or incinerators, such waste also constitutes an underutilized resource. Given that the mesocarp of passion fruit is rich in pectin, comprising approximately 25% of its composition, it offers considerable potential for value-added utilization.^4^ Therefore, the extraction and commercialization of pectin from passion fruit peel represent a viable approach that can enhance economic efficiency, improve sustainability, and reduce agricultural waste.

Pectin is a complex polysaccharide rich in (1→4)-α-linked d-galacturonic acid units as a homogalacturonan backbone with rhamnogalacturonan side chains of branched neutral sugars (such as rhamnose, arabinose, and galactooligosaccharides).^4,5^ Some of the carboxyl groups of galacturonic acid are partially methyl esterified, which gives the molecules a specified degree of hydrophobicity.^4^ Depending on the plant source and the extraction method, galacturonic acids can be partially or extensively methyl esterified. This amount of methoxyl groups is used to categorize pectin into high-methoxyl (degree of methyl esterification >50%) and low-methoxyl (degree of methyl esterification <50%) pectins.^4^ Conventional industrial pectin extraction processes typically employ a variety of acids, such as citric acid,^6,7^ tartaric acid,^8^ acetic acid,^8^ hydrochloric acid,^9,10^ and nitric acid.^11,12^ The type and concentration of acid are the primary factors influencing the yield, methyl esterification degree, molecular weight, and physical properties of pectin. Pectin derived from passion fruit peel through conventional acidic extraction is usually the high-methoxyl type^7,9,13^ with the molecular weights in the range of 100–250 kDa.^4^ The highest pectin yield was obtained from nitric acid extraction; meanwhile, the pectin extracted from citric acid was firmer than the others.^7^ This may be because of higher methyl esterification and molecular weight, which are influenced by the feedstock, medium pH, temperature, and extraction time.^4^ Following the 12 principles of green chemistry reported by Paul Anastas & John Warner,^14^ sustainable solvents ought to be employed with the expectation that they will not have a detrimental environmental impact. Citric acid, a weak organic acid found in lemons and limes, is readily soluble in water and biodegradable. Therefore, it is commonly utilized as a more ecologically friendly alternative to inorganic acids. Furthermore, pectin is already approved as a Generally Recognized As Safe (GRAS) food additive by the FDA.^15^ Therefore, it enjoys an increase in popularity because it is considered a potential alternative to commodity plastics in the food packaging field.^16^ Nonetheless, plant-extracted pectin films have poor mechanical strength, flexibility, moisture, and water-barrier qualities due to their hydrophilicity.^17^ In this regard, the aforementioned limitations can be overcome by incorporating natural polymers, such as chitosan,^6,18^ alginate,^19,20^ whey protein,^21^ starch,^22,23^ and xanthan gum^24^ into pectin-based films to achieve the desired properties.

Agarose, a natural polysaccharide with hydrophobic, biocompatible, nontoxic, and biodegradable nature, is an unbranched polysaccharide with repeating disaccharide units of β-d-galactose (1→3 linkages) and 3,6-anhydro-α-l-galactose (1→4 linkages).^25^ It is well known for its excellent gelling capability, forming stable thermoreversible gels, and its ability to reinforce polymeric matrices.^26^ These properties render agarose a valuable additive for enhancing the structural integrity, water-resistance, and mechanical performance of pectin-based films. In the literature review, the incorporation of agar into pectin has been reported to improve the tensile strength, stiffness, moisture content, and solubility of the resulting films compared to pectin films alone.^27^ Existing publications on pectin–agar composites primarily focus on developing active functional packaging films by incorporating various ratios of plant extracts^27^ or nanoparticles (*e.g.*, melanin^27^ and zinc sulfide^28^). In these studies, the film formulations relied on a fixed solid ratio (2 g pectin and 2 g agar^27,28^), and the pectin used was commercial citrus-derived powder. To date, no study has systematically examined how agarose source influences the physical properties of plant-extracted pectin films. Accordingly, identifying an optimal agarose loading to mitigate the inherent limitations of pectin films is warranted.

Raising from aforementioned concerns, this study is the first to (1) investigate the extraction conditions of pectin from purple passion fruit mesocarp (PPMP) using the eco-friendly citric acid method and (2) evaluate how different agarose concentrations (0.5%, 1.0%, and 1.5%) affect the physical properties of the resulting films, and (3) apply these pectin–agarose coatings to fresh bananas and assess their preservation performance. We seek to upgrade the value of passion fruit mesocarp and extend its use in food-related platforms and products. The novelty of this work is the use of PPMP, modified with agarose across a range of weight ratios to overcome the known limitations of plant-derived pectin material. In addition, the beneficial effects of PPMP/agarose coatings on the physicochemical properties of postharvest bananas have not been comprehensively reported. More importantly, the proposed banana-preservation coatings based on PPMP and agarose meet green and circular-economy criteria by addressing environmental pollution and converting agricultural residues into value-added packaging materials.

## Results and discussion

### Effect of condition parameters on pectin extraction and pectin characterization

The main paragraph text follows directly on here. The effects of conditional factors, including peel powder size, powder : extraction solvent ratio, temperature, time, and ethanol concentration used for pectin precipitation, on the extraction efficiency of PPMP are shown in Fig. B1. The results exhibited that the PPMP yield is 29.83 ± 0.63%, obtained under the optimal conditions as follows: powder size of 0.25 mm, mesocarp powder-to-citric acid ratio of 1 : 25 g mL^−1^, temperature of 90 °C, time of 60 min, and ethanol concentration used for pectin precipitation of 96%. Given the polysaccharide nature of purple passion peels, the PPMP was extracted with a great yield, demonstrating the efficiency of the adopted process. This value was higher than that reported by Nguyen *et al.*^6^ with extraction yields for purple passion fruit peel of 16.16% and Kulkarni *et al.*^29^ with the productivity of 14.80% for yellow passion fruit peel. Although response surface methodology (RSM) can provide comprehensive interaction analysis, the present study employed a one-factor-at-a-time (OFAT) approach for preliminary parameter screening. The focus of this work was not exhaustive statistical optimization but the establishment of suitable extraction conditions for subsequent material development. Future studies may incorporate multivariate optimization to further refine the extraction process.

ATR-FTIR spectra were utilized to determine the primary functional groups in the pectin structure isolated from purple passion fruit mesocarp (Fig. B2). The presence of the absorption band around 3600–3000 cm^−1^ due to O–H stretching vibrations was assigned to intra- and inter-molecular hydrogen bonds.^30,31^ A low transmittance peak at 2935 cm^−1^ is assigned to C–H stretching and bending vibrations.^32,33^ A characteristic peak appeared at 1720 cm^−1^, attributed to the absorption of the methyl esterified carboxylic group (–COO–R) present in the galacturonic acid chain characteristic of the pectin backbone,^30,34^ and the intensity at 1655 cm^−1^ corresponded to the asymmetric and symmetric stretching of the carboxylate group (–COO).^31^ The peak at around 1220 cm^−1^ suggested the existence of the –O–CH_3_ group.^33^ This spectrum also shows the “fingerprint region” of polysaccharides between 1100 cm^−1^ and 1000 cm^−1^, which is consistent with the structure of pyranose and furanose.^30,35,36^ In addition, the two characteristic bands at 920 cm^−1^ and 829 cm^−1^ were related to the absorption of β-d-glucopyranosyl and α-d-mannopyranose.^35,37^ These findings indicated that furanose and α-,β-pyranose rings existed in the pectin of passion fruit peel.^30^ The methyl esterification degree (DE) of PPMP was determined based on the ratio of the area of the esterified group (1720 cm^−1^) to the sum of the areas of the esterified group and the free carboxylic group (1720 cm^−1^ and 1655 cm^−1^).^34^ The DE for the as-prepared PPMP was more than 66.79%, which is categorized as high methoxy pectin (DE > 50% (ref. 38)). The DE in this work is higher than that of yellow passion fruit reported in the previous literature using citric acid.^6,7,13^ This may be due to the difference in passion fruit type, maturity, and extraction conditions. Besides, the PPMP was characterized in terms of its moisture of 3.66 ± 0.29% and ash contents of 8.70 ± 0.01%, based on the dry weight of matter and DPPH˙ free radical scavenging capacity of 11.86 ± 0.41%.

### Film characteristics

#### Attenuated total reflectance Fourier-transform infrared analysis (ATR-FTIR)

**Fig. 1:**
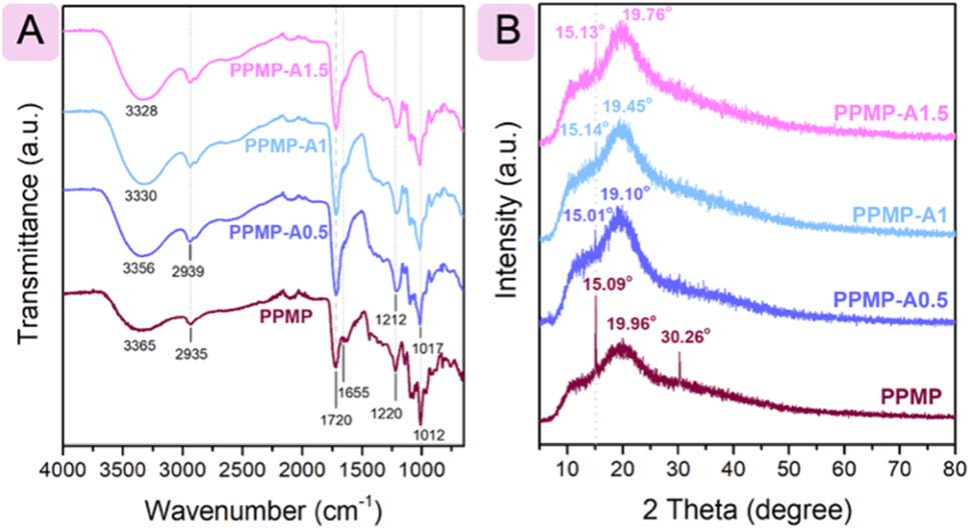
ATR-FTIR (A) and XRD (B) of PPMP and blend films.

ATR-FTIR analyses were conducted to determine the functional groups in the blend films and to investigate the possible interactions between agarose and PPMP. Fig. 1A illustrates the spectra of the PPMP films after being blended with agarose at various ratios. For the ATR-FTIR of PPMP/agarose blend films, the individual peaks of agarose were observed besides the typical peaks of PPMP. For instance, 1642 cm^−1^ was recognized as the stretching vibration of the conjugated carbonyl group in agarose, and 2884 cm^−1^ was due to the C–H stretching vibration of the methyl groups of the agarose polymer chain.^27,28^ Compared with the parent PPMP film, the ATR-FTIR of the blend film shows slightly higher band intensity in the 3400–2700 cm^−1^ and 1100–900 cm^−1^ regions, and a lower overall intensity between 1700–1200 cm^−1^. Furthermore, of particular interest is the observation that some changes in the peak positions in the spectra of PPMP film were identified after blending with agarose. In detail, peaks at 3365 cm^−1^ (O–H stretching vibration), 2935 cm^−1^ (C–H vibration), 1220 cm^−1^ (C–O group), and 1012 cm^−1^ (C–O–C stretch) were shifted to 3356–3328 cm^−1^, 2939 cm^−1^, 1212 cm^−1^, and 1017 cm^−1^, respectively. These peak movements likely suggest the intermolecular interactions, such as van der Waals and the hydrogen bonding among the agarose ether linkages (C–O–C), hydroxyl groups (–OH) of PPMP and glycerol.^18^

#### X-ray diffraction (XRD)

The structural characteristics of the films were investigated using XRD. The neat PPMP film displayed a broad diffraction peak centered at approximately 2*θ* = 19.96° (Fig. 1B), which is characteristic of the amorphous regions of pectin.^39–41^ Moreover, the diffraction peaks observed at 15.09° and 30.26° indicate the existence of a crystalline fraction within the pectin matrix, in agreement with earlier reports.^39–41^ After agarose incorporation, the diffraction peak intensity of the resulting films increased around 2*θ* ≈ 19.96°, which may be attributed to overlap with the semi-crystalline peak of agarose.^42,43^ Of particular interest is the observation that the peaks at ∼15° and 30.26° observed in the original PPMP film became weaker and even disappeared, respectively, after agarose addition. This behaviour suggests that even a small amount of semi-crystalline agarose can disrupt the ordering and continuous chain–chain interactions in the PPMP matrix, replacing them with saccharide–saccharide intermolecular interactions. This interpretation is consistent with the reduced fracture strength observed for PPMP-A0.5 and PPMP-A1. Simultaneously, the main diffraction peak shifted from 19.96° in PPMP to 19.10° in PPMP-A0.5, and then slightly increased to 19.45° in PPMP-A1 and 19.76° in PPMP-A1.5. This peak shift indicates the successful incorporation of agarose into the pectin matrix. At higher agarose contents, stronger intermolecular interactions, particularly hydrogen bonding as evidenced by ATR-FTIR analysis, may promote partial rearrangement of the polymer chains, leading to the slight shift of the peak toward higher angles. Furthermore, the absence of additional sharp peaks in the diffraction patterns confirms that the as-prepared PPMP films were free of impurities. Overall, the incorporation of agarose alters the structure of pectin, which in turn significantly influences the physicochemical properties of the films discussed in the following sections.

#### Visual appearance and color parameters

Table 1 depicts visual images of blend films made from the coating solution after drying. All prepared films had a smooth, glossy, uniform surface with an extremely transparent appearance. The PPMP film is light brown, but the PPMP/agarose films tend to turn light yellow, exhibiting a dose-dependent effect of agarose. A significant difference (*p* < 0.05) in the film color parameters, ruling out *a* value, is also summarized in Table 1. In detail, there is no significant difference (*p* < 0.05) in *L* value between parent PPMP and PPMP-A0.5 films, whereas PPMP-A1 and PPMP-A1.5 are much lighter. In contrast, *b* values increased progressively from 13.46 to 17.12, indicating a stronger yellowness of PPMP-A1 and PPMP-A1.5. The total color difference (Δ*E*) reveals that PPMP-A0.5 differs only slightly from the PPMP (Δ*E* = 2.42), while PPMP-A1 and PPMP-A1.5 show more noticeable differences (Δ*E* = 4.61 and 7.57, respectively). Overall, adding agarose progressively enhances the lightness and yellowness of blend films. In summary, these findings are consistent with the representations in the visual images.

**Table 1:** Visual image and properties of PPMP/agarose films^a^

	PPMP	PPMP-A0.5	PPMP-A1	PPMP-A1.5
Visual image	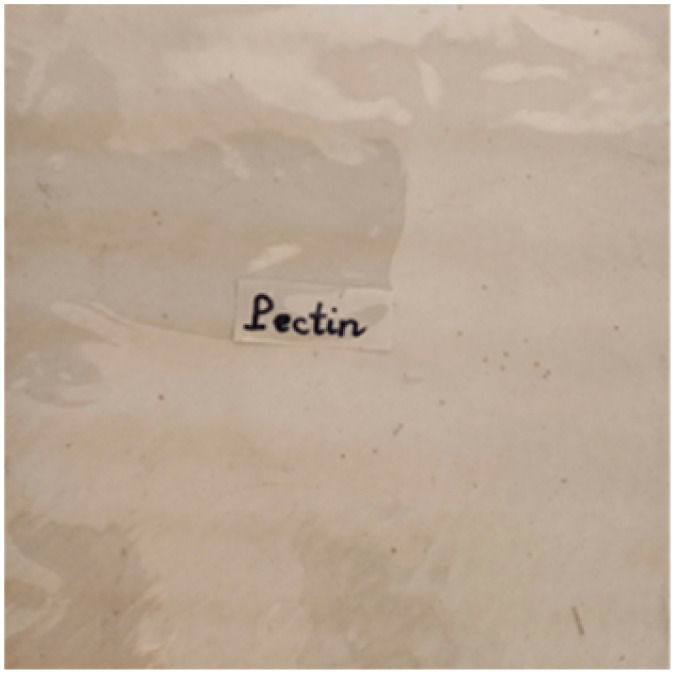	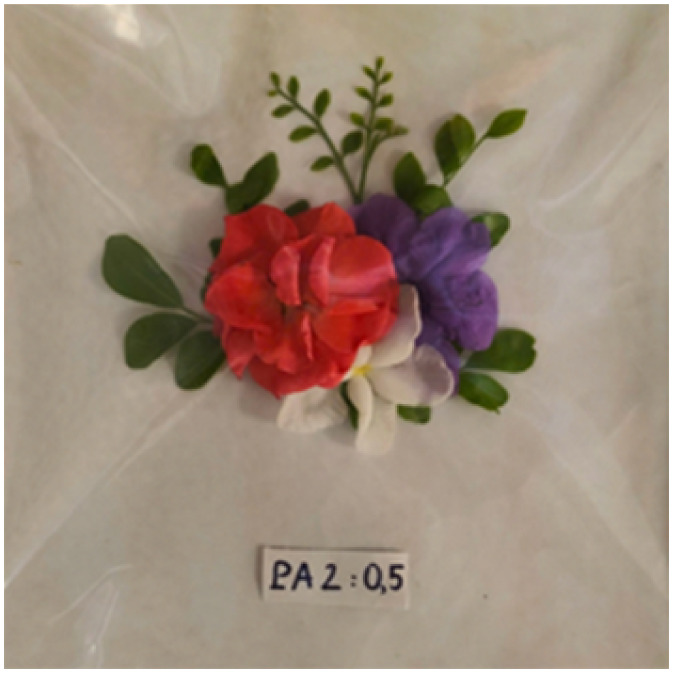	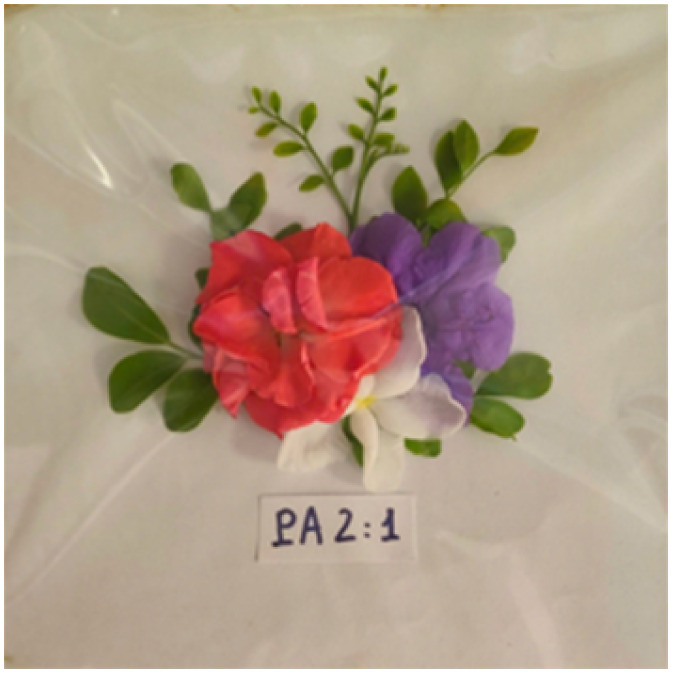	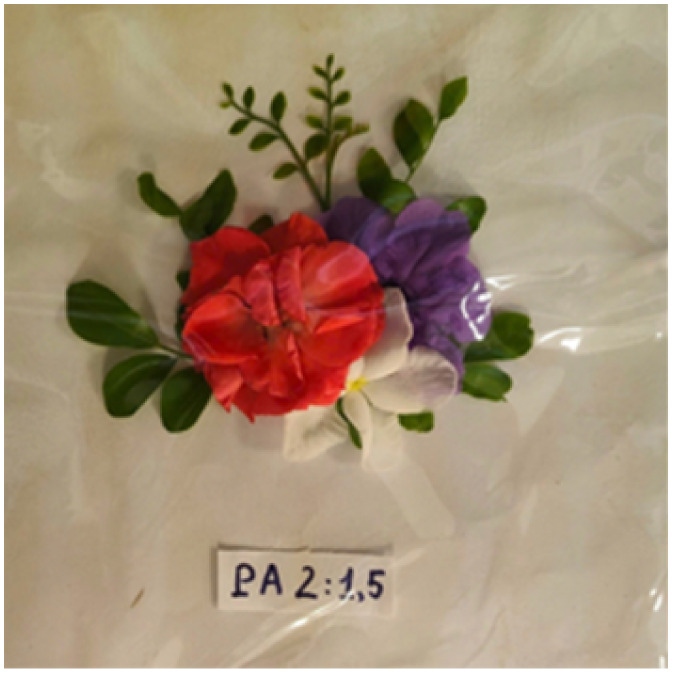

**Color parameters**				
*L*	78.48^a^ ± 0.29	78.39^a^ ± 0.31	81.24^b^ ± 0.47	85.03^c^ ± 0.69
*a*	0.76^a^ ± 0.06	0.59^a^ ± 0.08	0.42^a^ ± 0.03	0.33^a^ ± 0.07
*b*	13.46^a^ ± 1.00	15.84^ab^ ± 0.07	17.07^b^ ± 0.64	17.12^b^ ± 0.75
Δ*E*	—	2.42^a^ ± 0.66	4.61^b^ ± 0.40	7.57^c^ ± 0.52
Thickness (µm)	85.67^a^ ± 3.23	105.56^b^ ± 0.79	111.33^c^ ± 1.63	126.11^d^ ± 1.34

a Different letters within each row indicate significant differences according to Tukey’s HSD test (*p* < 0.05).

The film thickness is significantly affected by the solid content within the film matrix. As detailed in Table 1, the thickness of the PPMP film increases significantly (*p* < 0.05) with increasing agarose content. Given that the addition of agarose increased the solids content of the film-forming solution, resulting in a thicker film after drying. A similar phenomenon was observed in the agar-loaded commercial pectin film.^27^

#### Water susceptibility

**Fig. 2:**
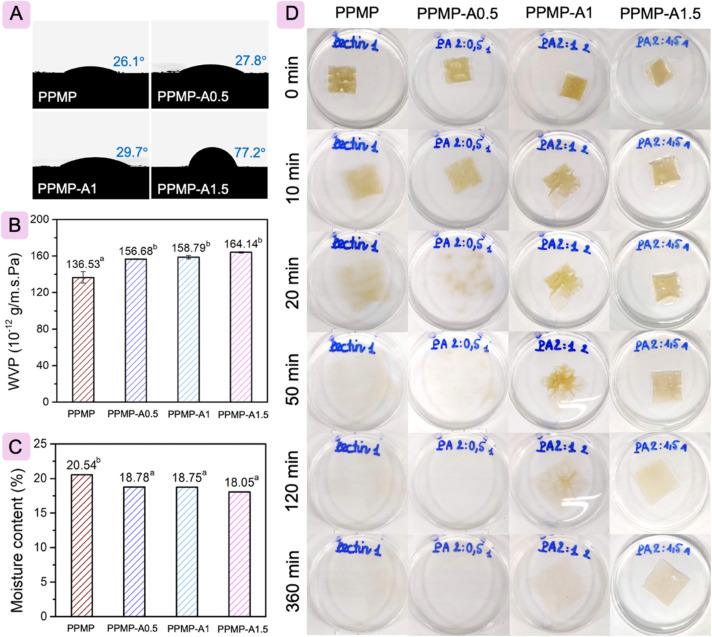
Water contact angle (A), water vapor permeability (B), moisture content (C), and the dissolution and swelling conditions (D) of all films.

The surface hydrophobicity/hydrophilicity of PPMP and PPMP/agarose films was estimated by determining the contact angle (CA) of a water droplet on the films' surfaces, as presented in Fig. 2A. The control PPMP film has a CA of 26.1°, suggesting a hydrophilic nature. This outcome was comparable with previously reported values for red dragon fruit peel-extracted pectin films.^44^ The PPMP/agarose blends exhibit higher CA than neat PPMP film, with 1.5% agarose yielding the greatest increase (77.2°). Similar behavior was reported on the addition of agar to gelatin film^45^ and commercial citrus pectin film,^27^ with enhanced CA values corresponding to an increase in agar concentration. The enhancement in CA of PPMP/agarose films could originate from the diversification in the amount of hydrophobic and hydrophilic groups within the film network. The film surface is classified as hydrophobic when the CA is more than 65°,^46^ implying that the agarose supplementation at a 2 : 1.5 (w/w) ratio increased the hydrophobic property of the neat PPMP film. Given that a decrease in the number of exposed hydroxyl groups in PPMP and hydroxyl groups in agarose within the blend films is driven by intermolecular interactions between PPMP and agarose, as compared to the individual components.^47^

**Fig. 3:**
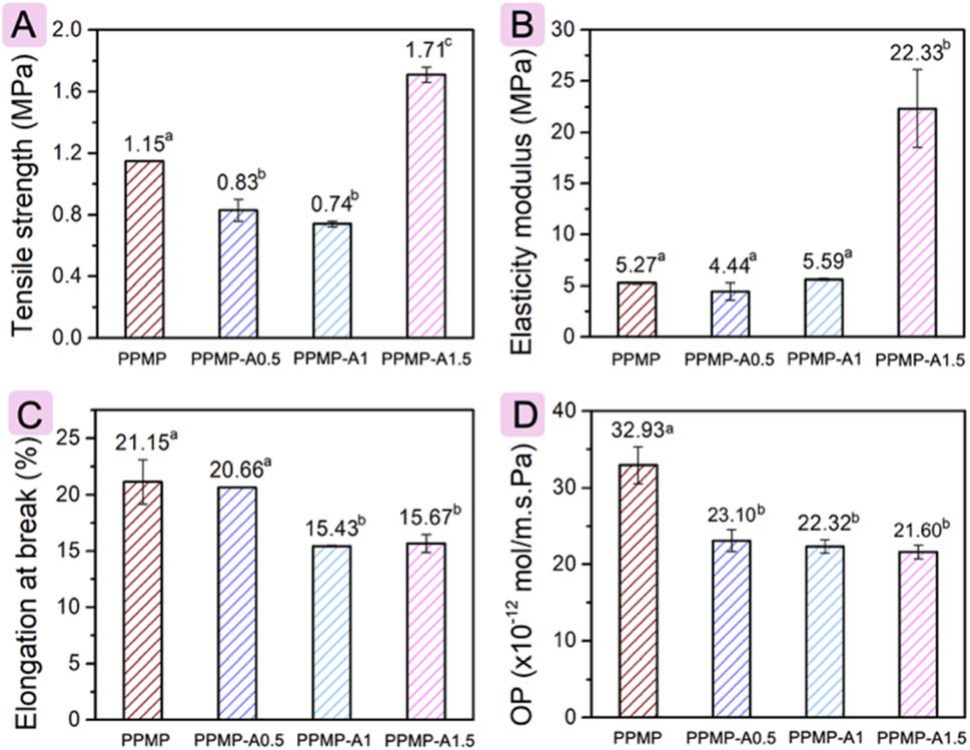
Tensile strength (A), elasticity modulus (B), elongation at break (C), and oxygen permeability (D).

Water vapor permeability (WVP) reflects the absorption and diffusion of water vapor across the film matrix, and the WVP values of the film are shown in Fig. 2B. The WVP value increases significantly with the addition of agarose, but it does not show a dose-dependent effect of agarose, ruling out PPMP-A1.5. As reported, film thickness and the proportion of hydrophilic and hydrophobic groups are closely related to the WVP of hydrophilic films.^48–50^ In brief, film thickness below 0.030 mm has a significant impact on the WVP value.^47^ Consequently, the WVP of the neat PPMP film slowly increases with the addition of agarose. Notably, the PPMP-A0.5, PPMP-A1, and PPMP-A1.5 films showed comparable WVP values, indicating that, beyond film thickness, the internal structure of the films also significantly influences water vapor permeability in hydrophilic systems. These results suggest that varying the agarose content did not significantly modify the intrinsic water vapor barrier characteristics of the polymer matrix. This phenomenon may be attributed to a compensatory effect between diffusivity and sorption mechanisms within the film matrix. Accordingly, the PPMP-A1.5 exhibited higher TS and EM with reduced EaB (Fig. 3), indicating a more compact and rigid network that could restrict water molecule diffusion. However, the PPMP-A1.5 film maintained a comparable WVP value as the other blend films regardless of film thickness, which may be associated with some free hydroxyl groups in both PPMP and agarose that are available to interact with water molecules.^47^ The increased CA observed for PPMP-A1.5 reflects changes in surface wettability rather than bulk transport properties. Since WVP is determined by diffusion through the entire film thickness rather than surface characteristics alone, the overall water vapor barrier performance remained statistically unchanged. Similar behavior was reported on the addition of agar into neat commercial citrus pectin films, with a significant increase in the WVP value of the resultant film.^27^ Regarding the moisture content (MC), the blend film exhibits a lower MC compared to the pure PPMP film (Fig. 2C). This decrease can be attributed to the intermolecular hydrogen bonding between the surface hydrophilic hydroxyl groups of agarose, pectin, and glycerol within the film network.^51^ Roy and Rhim^27^ also reported lower MC values of commercial citrus pectin/agar films than films containing only agar or pectin. The reduction in MC of the pectin-based blend film is favorable for food packaging, since lower moisture improves dimensional stability and reduces microbial susceptibility, thereby enhancing the shelf life of food products. However, there was no dose-dependent trend of agarose on the MC of the blend film.

Fig. 2D visually depicts the solubility and swelling behavior of the films when immersed in water for a certain time. All films wrinkled upon initial contact with the aqueous solution. After 20 min, the PPMP film and the PPMP-A0.5 blend film disintegrated entirely, while the other two integrated films retained their structures. Following a 360 min exposure, only the PPMP-A1.5 film retained its integrity. These observations are consistent with the CA results. This demonstrates that a higher agarose content improves cross-linking within the film network, resulting in a tighter matrix structure with fewer free polar groups on the surface that may directly contact water, thereby enhancing the film's integrity.

#### Mechanical behavior

The influence of agarose on the mechanical behavior of films was assessed through tensile strength (TS), elongation at break (EaB), and elastic modulus (EM), as displayed in Fig. 3A–C. The PPMP-A1.5 film exhibits the highest TS and EM. Compared to the parent PPMP film (1.15 MPa in TS and 5.27 MPa in EM), the TS and EM of the PPMP-A1.5 film are significantly improved by over 48% and 323%, respectively (*p* < 0.05). However, the PPMP-A0.5 and PPMP-A1 films showed a significant decrease (*p* < 0.05) in TS, while their EM values are insignificantly different (*p* < 0.05) among film formulations. Given that a small amount of semicrystalline agarose can interrupt local ordering and continuous chain–chain interactions in PPMP, lowering the tensile strength of blend films. However, a higher concentration of agarose (1.5% w/w based on PPMP weight) yielded a significant increase in TS and EM of the PPMP-A1.5 film. This increment is likely due to good compatibility between agar and pectin.^52^ On the other hand, linear agarose molecules may serve as reinforcing agents when properly dispersed in pectin, which intercalate into the pectin network and form additional hydrogen-bonded junctions, thereby enhancing the film's stiffness and tensile strength.^27^ The EaB of PPMP and PPMP-A0.5 shows an insignificant difference. However, further increasing the agarose content caused a significant reduction (*p* < 0.05) in the EaB of PPMP-A1 and PPMP-A1.5. This aligns with the TS and EM because adding a higher content of agarose increases film stiffness and fracture strength, but decreases flexibility by restricting the macromolecular chain movement of pectin during deformation. Additionally, the PPMP films exhibited a higher internal moisture content than the blend films. As a result, the absorbed water plasticizes the polymer matrix, reduces intermolecular interactions, and thereby enhances flexibility and chain mobility. Similar behavior in EB decrease was reported on adding agar to the commercial citrus pectin,^27^ potato starch,^53^ and fish gelatin^54^ matrices.

#### Oxygen permeability

Oxygen markedly affects food quality and shelf life by accelerating oxidation and spoilage.^55^ Pectin films provide limited resistance to oxygen permeation due to their hydrophilicity, and their barrier efficacy declines with rising relative humidity as absorbed water plasticizes the matrix and increases gas diffusivity. Meanwhile, agarose films provide comparatively strong barrier performance owing to their stiff, stable network.^56^ Herein, the oxygen permeability (OP) values of the PPMP, PPMP-A0.5, PPMP-A1, and PPMP-A1.5 films are 32.93 × 10^−12^ mol m^−1^ s^−1^ Pa^−1^, 23.10 × 10^−12^ mol m^−1^ s^−1^ Pa^−1^, 22.32 × 10^−12^ mol m^−1^ s^−1^ Pa^−1^, and 21.60 × 10^−12^ mol m^−1^ s^−1^ Pa^−1^, respectively (Fig. 3D). This pattern underscores the critical role of adding agarose in improving the oxygen barrier performance of the PPMP film. This effect is likely attributed to the increased film thickness and the formation of a cross-linked agarose–PPMP network, which stiffens the films and impedes oxygen diffusion through the matrix. Because OP depends strongly on film water content,^57^ the lower intrinsic moisture of the PPMP/agarose films results in reduced oxygen transmission compared with parent PPMP films. These findings suggest a potential benefit for extending fruit shelf life in subsequent studies. However, no dose-dependent trend in the OP value of the blend films made from PPMP and agarose, ruling out PPMP-A1.5, is due to their similar thickness.

### Banana preservation

#### Microscopic characterization of coated banana peel

**Fig. 4:**
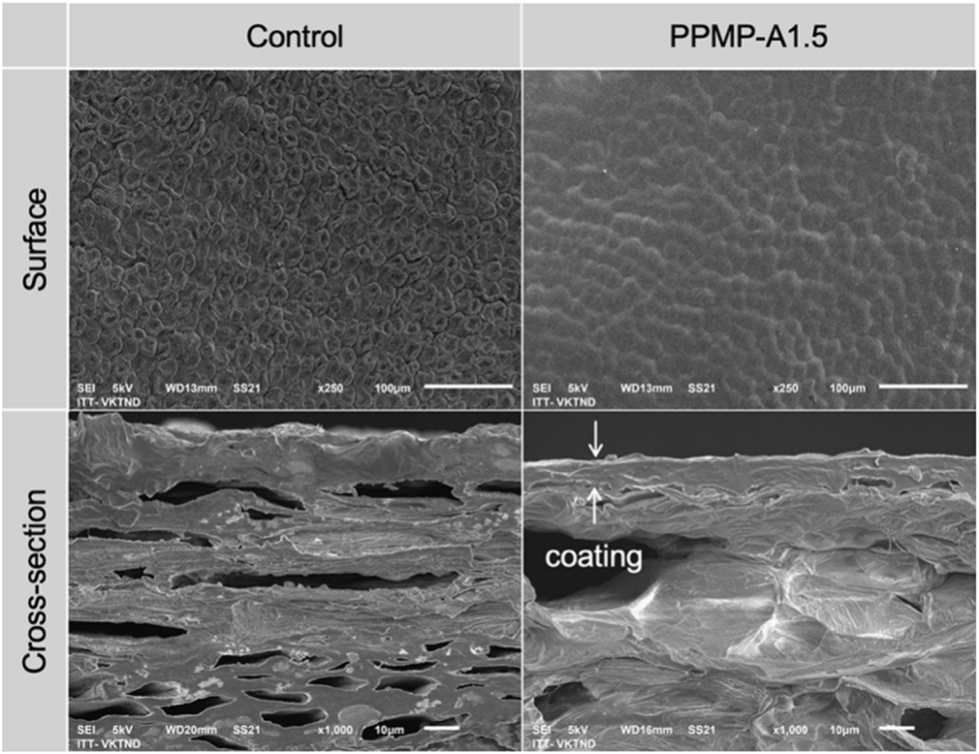
Surface and cross-sectional morphology of uncoated and coated banana peel.

To better understand the formation of the coating layer on banana peel, SEM was performed to investigate the surface morphology and internal structure of uncoated and PPMP-A1.5-coated banana peels (Fig. 4). The uncoated sample exhibits a rough, heterogeneous stomatal structure with clearly visible epidermal cells. In contrast, the PPMP-A1.5-treated peel shows a noticeably smoother and more matte surface. The underlying stomatal structure is largely masked by a continuous thin layer, suggesting successful formation of a protective coating over the peel surface after being dipped in PPMP-A1.5 solution. Cross-sectional observations further confirm these differences. The control sample exhibits an uneven and irregular top-edge, while the PPMP-A1.5-treated sample presents a flat and smooth top-edge with a continuous coating layer adhered to the outer peel surface (indicated by arrows). The coating appears compact and well-integrated with the peel tissue, without obvious cracks or delamination. The coating thickness, roughly estimated from the SEM micrograph to be 5–8 µm, confirms the successful deposition of a coherent coating layer. This finding supports the interpretation that the improved preservation performance is associated with enhanced surface barrier properties, which help inhibit respiration, reduce weight loss, and slow softening.

#### Change in visual appearance and peel browning

**Fig. 5:**
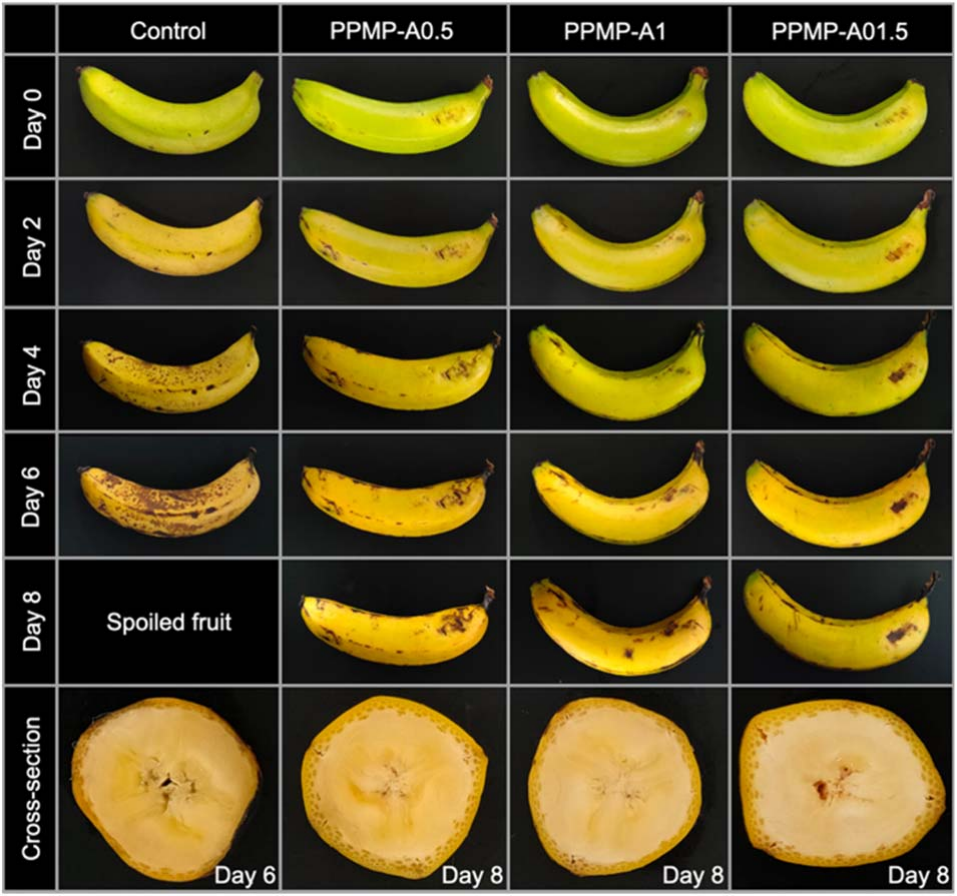
Visual appearance of the uncoated and coated bananas during storage.

**Fig. 6:**
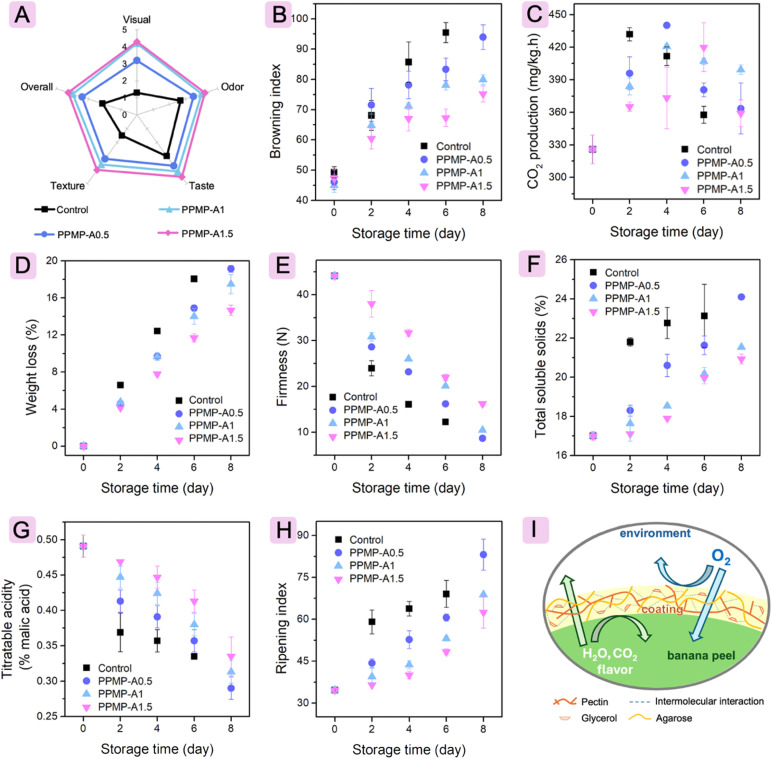
Sensory evaluation (A), and changes in browning index (B), weight loss (C), firmness (D), total soluble solids content (E), titratable acidity (F), ripening index (G), and pH (H) of banana during storage, and possible mechanism of banana preservation using PPMP-A1.5 coating (I).

The preservation effect of PPMP/agarose coatings with different agarose ratios on fresh bananas is shown in Fig. 5. At the beginning of storage, the external appearance of all bananas is yellow with some green at stage 3 ripeness.^58^ In general, the PPMP/agarose-coated groups appear glossy to the naked eye, revealing that PPMP/agarose coatings have a positive impact on the fruit's appearance. The control group exhibits a yellow-brown color shift, accompanied by random black spots that emerged drastically after 4 days of storage. These changes are attributed to the faster cell respiration rate induced by the formed ethylene gas as a characteristic of climacteric fruit.^59^ Surprisingly, bananas in the PPMP-A1.5 group ripen more slowly up to the 8^th^ day. Particularly, the ripening stage of PPMP-A1.5-coated fruit on day 8^th^ is comparable to that of PPMP-A1-coated fruit on day 6^th^, PPMP-A1.5-coated fruit on day 4^th^, and uncoated fruit on day 2^nd^. Furthermore, a panel of 50 trained participants completed a survey to rate the preservative performance of the coatings on bananas on the 6^th^ day of storage based on their color, texture, taste, odor, and overall impression. The results present that the scores for the bananas coated with PPMP-A1.5 are significantly higher than those in the other groups (Fig. 6A). These outcomes demonstrate that the PPMP-A1.5 coating formulation is more effective in preserving bananas compared to the other groups.

The changes in browning index (BI) of bananas are observed during the storage period, as seen in Fig. 6B. Statistical analysis shows that the coating formulation and storage time have a noteworthy effect (*p* < 0.05) on the development of brown spots on the banana peel (Table B1). In general, no noticeable differences in BI values are observed among the groups during the first 2 days, followed by a significant increase in the subsequent days of storage. The increment in the BI with the extension of the storage period is due to the progressive enzymatic browning and tissue degradation that occur during ripening and senescence.^60^ Disruption of banana cell structure brings polyphenol oxidase (PPO), phenolic substrates, and oxygen into contact. PPO catalyzes their oxidation to *o*-quinones; these reactive intermediates undergo non-enzymatic polymerization and condensation, yielding brown melanin on the peel surface.^61^ There are significantly different behaviors in the BI value of the uncoated and coated groups. The BI value of the control group increases markedly within 6 days of the holding period, which corresponds to the visible appearance of dark spots and browning. The control fruit presents a significantly higher BI value than the PPMP-A1.5- and PPMP-A1-coated fruit on day 4^th^ and day 6^th^, whereas an inconsequential difference is observed between the control and the PPMP-A0.5-coated fruit. For treated groups, the BI value of the PPMP-A1-coated and PPMP-A1.5-coated bananas changes more slowly during storage compared with the PPMP-A0.5 group. On the 8^th^ day, the BI of the fruit treated with PPMP-A0.5 is much higher than that of the fruit with the PPMP-A1 and PPMP-A1.5 coatings, consistent with visual appearance. The lowest change in BI value is observed in the PPMP-A1.5 group, indicating the banana preservative efficiency of this coating. This is likely because the PPMP-A1.5 coating has an effective water and oxygen barrier, thereby slowing down the auto-oxidation activity of PPO, which catalyzes the conversion of phenolic compounds to quinones that subsequently polymerize into melanins, leading to browning of the banana peel.^62^ Furthermore, there is a nonsignificant interaction in the BI value between holding period and different coatings.

#### Change in respiration rate, weight loss, and firmness

The respiration rate, used as an indicator to assess the preservation effect of the PPMP/agarose coatings on bananas, is determined by measuring the amount of CO_2_ released during storage. Storage time has a substantial effect (*p* < 0.05) on the amount of CO_2_ produced from all groups (Table B1). The CO_2_ production from the bare group sharply increases from the first day and peaks on day 2^nd^ (432.06 mg kg^−1^ h^−1^) before decreasing sharply in the following days of storage, as illustrated in Fig. 6C. Fruit coated with PPMP-A0.5 and PPMP-A1 coatings hit the highest point on day 4^th^ (440.17 mg kg^−1^ h^−1^ and 420.72 mg kg^−1^ h^−1^, respectively), while the PPMP-A1.5-coated fruit reached the peak at day 6^th^ (419.97 mg kg^−1^ h^−1^). It can be seen that uncoated bananas have the highest CO_2_ production rate, while the lowest amount of CO_2_ is found in PPMP-A1.5-coated bananas. The respiration process utilizes O_2_ from the surrounding environment to produce more CO_2_ as follows: C_6_H_12_O_6_ + 6O_2_ ⟶ 6CO_2_ + 6H_2_O + Energy.^63^ As mentioned above, the PPMP-A1.5 film possessed the lowest oxygen and water permeability among film formulations; thus, this coating restricts the oxygen supply to the respiration process, thereby slowing the metabolism and ripening rate of the fruit. Table B1 exhibits that the different coatings have an insignificant influence (*p* > 0.05) on the respiration rates of bananas during the duration of storage. Also, the statistical interaction between various coatings and storage periods is found to be non-significant.

Fig. 6D illustrates the percentage weight loss of all groups during storage times. The holding period has a significant influence (*p* < 0.05) on the weight loss of banana groups during preservation (Table B1). In general, the weight loss rate of bananas rises steadily as the storage duration is extended. Weight loss increases in all groups owing to water loss and nutrient consumption.^62^ Water evaporation is primarily driven by transpiration through the stomata in the epidermal cells, while nutrients are consumed in the metabolic processes of the fruit.^64^ Furthermore, Tukey HSD statistical evaluation reveals that the weight loss of bananas during the holding period is considerably impacted (*p* < 0.05) by different coating formulations. Specifically, the uncoated group shows higher weight loss than the treated groups throughout the storage period. There is a substantial difference owing to the coating effect on day 2^nd^, but on subsequent days of storage, in which three groups are clearly identified. Fruit dipped in PPMP-A1.5 coating solution has a lower percentage of mass loss than those dipped with PPMP-A0.5 and PPMP-A1 during storage. No significant difference (*p* > 0.05) is observed in the weight loss between fruit coated with PPMP-A0.5 and PPMP-A1 during storage, ruling out the end of storage. On day 8^th^, the fruit treated with PPMP-A0.5, PPMP-A1, and PPMP-A1.5 exhibits a weight loss of 19.14%, 17.48%, and 14.67%, respectively. This is mainly because the PPMP-A1.5 coating has the greatest water and oxygen barrier, which reduces the respiration rate of bananas during storage and consequently slows their dehydration.

Variations in pulp firmness serve as a key indicator of fruit quality throughout storage. This index is closely related to water loss and metabolic changes.^65^ The storage period has a substantial effect (*p* < 0.05) on the pulp firmness (Table B2). Fig. 6E shows that the firmness values gradually decreased with increasing storage time. According to Xie *et al.*,^66^ ethylene released during banana ripening promotes the activity of pectinase and polygalacturonase, resulting in the degradation of pectin substances and the disruption of cell walls, resulting in a decrease in banana firmness. There is an obvious distinction in firmness between the untreated and coated groups (*p* < 0.05). The control group exhibits a faster declining trend in firmness with the extension of the storage time than the treated groups. Moreover, different coatings on the fruit have distinct behaviors in terms of firmness. The slower change in the firmness of fruit coated with PPMP-A1.5 is comparable to that observed in fruit coated with PPMP-A0.5 and PPMP-A1 during storage. In particular, the firmness of PPMP-A1.5-coated fruit at the end of storage is 16.22 N, higher than that of PPMP-A0.5-coated fruit (8.67 N) and PPMP-A1-coated fruit (10.46 N). This is primarily owing to the lowest oxygen and water permeability of the PPMP-A1.5 layer, which can seriously limit metabolism and delay the softening of the banana.

#### Change in total soluble solid content, titratable acidity, ripening index, and pH

The total soluble solids (TSS) content is a reliable indicator of fruit ripening, as starch is converted into soluble sugars such as glucose, sucrose, and fructose during the ripening process of bananas.^58^ Herein, the TSS is substantially affected (*p* < 0.05) by holding period and coating formulation (Table B2). There is a significant difference in TSS between treated and untreated bananas. The control group shows the fastest increase in TSS, while the slower changes in TSS are found in coated fruit (Fig. 6F). The coating effect on bananas is significantly different, as the PPMP-A0.5-coated group reveals a much faster rise in TSS value than the PPMP-A1- and PPMP-A1.5-coated fruit during storage, indicating that these two coatings are effective in retarding the ripening and metabolism of bananas. However, no significant interaction in the TSS between different coatings and storage periods is recorded.

Statistical analysis confirms the significant effects of storage time and coating formulation on the titratable acidity (TA) of bananas (Table B2). Generally, the TA percentage of all bananas gradually decreases as storage time increases (Fig. 6G). During fruit ripening, organic acids are often consumed through esterification or neutralization of alkaline compounds, which raises the pH and lowers TA.^67^ The downward trend in TA is in good agreement with changes in BI, weight loss, firmness, and TSS. Coating formulation significantly affected (*p* < 0.05) the fruit's TA. The uncoated group experiences a rapid decline in TA compared to the coated groups. Throughout storage, the PPMP-A1.5-coated group exhibits notably slower changes in TA compared to the PPMP-A0.5- and PPMP-A1-coated groups, demonstrating the preservative efficiency of the PPMP-A1.5 coating on fresh bananas. However, no statistical interaction between different coatings and storage periods in the TA is found.

Fig. 6H depicts the ripening index calculated from the ratio of TSS to TA. Statistical analysis confirms that storage periods and coating formulations affected the ripening index (*p* < 0.05). The ripening index increases for all groups with the extension of the holding period, regardless of the type of coating. The coating formulation has a major effect (*p* < 0.05) on fruit ripeness. A slower increase in ripening index is observed in coated bananas compared with untreated fruit. Similar to TA, the PPMP-A1.5-coated group exhibits notably slower changes in ripening than the PPMP-A0.5- and PPMP-A1-coated groups, suggesting its preservative performance on fresh bananas. Statistical interaction between different coatings and storage periods in the ripening rate has not been recorded.

ANOVA analysis shows that the effect of storage time and type of coating on the pH parameters of banana pulp is significant (*p* < 0.05), as presented in Table B2. The pH of all banana pulps increases steadily with storage time, in line with the downward trend of TA. As ripening progresses, consumption of organic acids *via* esterification and neutralization reactions reduces acidity and raises pH.^67^ The control exhibits a rapid increase in pH during storage, whereas the coating has a positive effect in slowing down the pH changes of fresh bananas. Various coatings behave differently when the fruit is coated with the PPMP-A1.5, slowly changing in pH than those treated with PPMP-A0.5 and PPMP-A1. This pattern underscores the critical role of PPMP-A1.5 coating in preserving fresh bananas. No significant interaction between coatings and storage periods has been documented. Compared to previous studies (Table 2), the PPMP-A1 coating was relatively effective in enhancing banana preservation but less effective than the chitosan and the carboxymethyl nanofiber coatings. This may be due to differences in coating composition, storage conditions, banana type, and harvest period.

**Table 2:** Biodegradable polymer-based packaging coatings for banana preservation

Formulation	Banana type	Result	Reference
Cornstarch/beeswax Pickering emulsion/cellulose nanocrystals	Yellow banana (Canada)	7 days at 20 °C	Trinh *et al.*^59^
Modified amphiphilic cellulose nanofibers/*n*-octyl succinic anhydride	Yellow banana (Finland)	6 days, not mention condition	Qasim *et al.*^68^
Carrageenan/sulfur quantum dots	Yellow banana (Vietnam)	8 days at 25 °C and 50% RH	Priyadarshi *et al.*^69^
Sodium alginate/whey protein isolate	Cavendish yellow banana (China)	6 days at 25 °C and 50% RH	Deng *et al.*^70^
Konjac glucomannan	Cavendish yellow banana (China)	7 days at 25 °C and 50% RH	Deng *et al.*^71^
Konjac glucomannan/xanthan gum	Cavendish yellow banana (China)	7 days at 25 °C and 50% RH	Deng *et al.*^72^
Microfibrillated cellulose	Yellow banana (USA)	6 days	Geng *et al.*^73^
Rice protein/sodium alginate emulsion film with oleic acid	Yellow banana (China)	4 days at 25 °C and 50% RH	Lou *et al.*^74^
Chitosan	Cavendish green banana (Vietnam)	10 days at 20 °C and 64% RH	Nguyen *et al.*^75^
Carboxymethyl cellulose nanofibers	Banana (Republic of Korea)	10 days	Kwak *et al.*^62^
Poly(vinyl alcohol)/agar/maltodextrin	Areca yellow banana (Vietnam)	5 days at 25 °C	Nguyen *et al.*^67^
Poly(vinyl alcohol)/agar/d-glucose	Grande Naine green banana (Vietnam)	6 days at 27 °C	Pham *et al.*^76^
Commercial citrus pectin/agarose	Chiquita green banana (Vietnam)	8 days at 25 °C and 64% RH	Pham *et al.*^77^
Purple passion fruit mesocarp extracted pectin/agarose	Cavendish green banana (Vietnam)	8 days at 25 °C and 64% RH	This work

#### Preservation mechanism

Fig. 6I illustrates the preservation mechanism of the PPMP-A1.5 coating in fresh banana. The PPMP-A1.5 coating inhibits the transfer of oxygen from the surrounding environment into the bananas, thereby reducing their metabolic processes. In addition, the PPMP-A1.5 coating preserves the desired freshness and texture of the fruit by decelerating water vapor diffusion, which in turn minimizes transpiration, weight loss, and softening. They also form a surface coating that helps prevent off-flavors and preserves the nutritional content of the bananas during storage.

## Experimental

### Chemicals

Agarose was procured from VWR Prolabo (Avantor, France). DPPH˙ free radical (1,1-diphenyl-2-picrylhydrazyl) was bought from Tokyo Chemical Industry Co., Ltd (Tokyo, Japan). Glycerol, citric acid monohydrate, and ethanol were purchased from Xilong Chemical Co., Ltd (Guangdong, China).

### Preparation of the passion fruit mesocarp powder

**Scheme 1:**
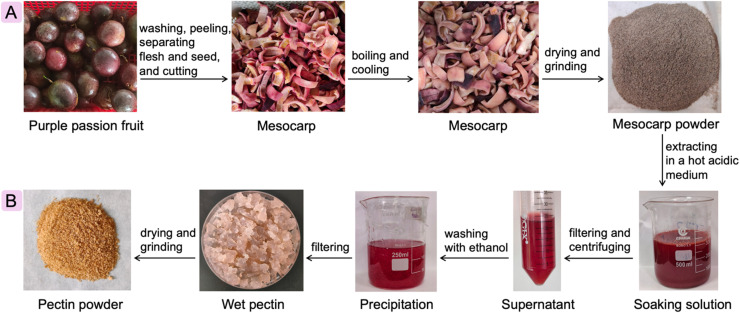
Process of pre-treatment (A) and the pectin extraction (B) from the passion fruit mesocarp powder.

Fresh purple-skinned passion fruit was bought from a farm in Bao Loc City, Lam Dong Province, Vietnam, on November 22^nd^, 2023, and delivered to the laboratory the next day. The passion fruit pre-treatment was carried out as described in Scheme 1A. The fruit was washed with tap water to remove dirt and sliced to separate the aril flesh and seed with the aid of a spatula. After the epicarp and endocarp of purple passion fruit were removed, the mesocarp (3–5 mm) was cut into small pieces and boiled in a water bath for 3 min, then blanched in an ice bath. Subsequently, the mesocarp was dried in an oven with air circulation at 60 °C for 72 h. The dried mesocarp was ground into powder using a domestic blender and filtered through a sieve. The obtained powder of passion fruit mesocarp with a moisture of 14.87 ± 0.55% and ash content of 9.46 ± 0.52% was packed in LDPE bags and stored in a dry and dark tin desiccator for further experiments.

### Pectin extraction and experimental design

Pectin was extracted from purple passion fruit mesocarp powder by the conventional method with citric acid as solvent (Scheme 1B). In detail, 4 g of dry powder was put into 100 mL of 1 M citric acid (pH 1.57) hot solution at 85 °C and stirred thoroughly for 90 min. Subsequently, the mixture was cooled and filtered through a filter cloth to collect the filtrate. The supernatant, after centrifugation at 5000 rpm for 5 min, was collected and precipitated with a double volume of 96% ethanol (supernatant : ethanol is 1 : 2 v/v) for 12 h. Subsequently, the precipitate obtained by filtration was washed three times with the 96% ethanol solution to remove soluble impurities and collect coagulated pectin. The wet pectin was dried at 50 °C for 24 h and ground to obtain PPMP powder, and then stored at room temperature in a cool and dry location.

In this study, one factor at a time was used to select the optimal process parameter levels for maximizing pectin yield, with one process parameter varied while the others remained constant. The parameters included dry mesocarp powder size (0.1, 0.25, and 0.315 mm), powder-to-citric acid ratio (1 : 15, 1 : 20, 1 : 25, and 1 : 30 g mL^−1^), extraction temperature (70, 80, 90, and 100 °C), extraction time (30, 45, 60, and 90 min), and ethanol concentration for pectin precipitation (75, 85, and 96%). All experiments were performed in triplicate to evaluate experimental error. The PPMP with the highest yield was further analyzed for methyl esterification degree, moisture content, ash content, and DPPH˙ free radical scavenging capacity before being used in film development. Details of the evaluation process are provided in Section A1.

### Film preparation

The PPMP/agarose films were facilitated by the film casting and solvent evaporation methods. Purple passion fruit mesocarp-extracted pectin solution (2%, w/v) was prepared by dissolving dried pectin powder in distilled water at 70 °C on a magnetic stirrer for 30 min. Agarose was added to the pectin solution at pectin to agarose ratios of 2 : 0.5, 2 : 1, and 2 : 1.5 (w/w). Subsequently, the pectin/agarose mixture was stirred at 70 °C for 1 h. The homogeneous mixture was plasticized with 30% glycerol (w/w relative to PPMP and agarose) and stirred continuously for 1 h at 70 °C. The film-forming solution was centrifuged at 6000 rpm for 1 min to eliminate air bubbles before being poured into a 20 × 20 cm polypropylene mold in the same volume to avoid variation among the films. After being dried at 60 °C for 72 h, the resulting film was peeled from the mold and stabilized in a desiccator for 24 h at 25 °C before further analyses.

Section A2 in the SI of the manuscript provides a detailed characterization of all films, including colorimetric measurement, thickness, attenuated total reflectance Fourier-transform infrared analysis, water contact angle, water vapor permeability, moisture content, mechanical testing, and oxygen permeability.

### Banana preservation

Fresh Dwarf Cavendish bananas (*Musa acuminata*, AAA group) were purchased from a garden in Dak Lak Province, Vietnam, on October 17^th^, 2024, and immediately transferred to the laboratory. In detail, a total of 240 bananas were carefully selected from the same bunch, washed with water, and dried naturally under a fan at 25 °C. The bananas were then randomly divided into 4 groups, including the uncoated group and three PPMP/agarose-coated groups (including PPMP-A0.5, PPMP-A1, and PPMP-A1.5). Briefly, the fruit were dipped in PPMP/agarose coating solution for 5 s, then placed on trays until dry, dipped twice more based on preliminary experiments, and further stored indoors at 25 °C and 64% RH. The untreated group was used as the control group. The microscope morphology of peel surface, along with sensory, weight loss, peel browning, hardness, total soluble solids content, titratable acidity, and pH of fruit, were tested every 2 days. The detailed evaluation procedure is presented in detail in Section A3.

## Conclusions

In conclusion, this study successfully develops an eco-friendly, biodegradable coating based on Vietnamese purple passion fruit mesocarps-extracted pectin (PPMP) and agarose for banana preservation. Among various PPMP/agarose ratios tested, the PPMP-A1.5 formulation showed the best performance, significantly improving the mechanical properties (fracture strength and flexibility), oxygen-barrier ability, and overall barrier resistance (including moisture content, contact angle, permeability, solubility, and swelling). In preservation tests, bananas coated with PPMP-A1.5 maintained better quality, showing less peel browning, reduced weight loss, preserved firmness, and slower changes in soluble solids, acidity, and pH as compared to other coatings. These results demonstrate that purple passion fruit peel is a valuable source of high-quality pectin suitable for industrial film production and highlight the potential of PPMP/agarose coatings as a sustainable method for extending the shelf life of fresh bananas. Future studies should focus on incorporating natural extracts into PPMP/agarose coatings to confer antioxidant and antibacterial properties and to evaluate their release in simulated food systems.

## Author contributions

Bao-Tran Pham-Tran: writing – original draft, formal analysis, methodology, data curation, visualization. Nhu-Quynh Thi Nguyen: formal analysis, validation, data curation. Nhu-Ngoc Quynh Nguyen: formal analysis, data curation. Long Giang Bach: validation, visualization, writing – review & editing. Thuong Thi Nguyen: writing – review & editing, methodology, investigation, validation, visualization, project administration, supervision.

## Conflicts of interest

There are no conflicts to declare.

## Supplementary Material

RA-016-D5RA09755J-s001

## Data Availability

The supporting data has been provided as part of the supplementary information (SI). Supplementary information: the influence of dry powder size, ratio of power : citric acid, temperature extraction, time extraction, and ethanol concentration used for pectin precipitation on the pectin extraction capacity (Fig. B1). FTIR of extracted pectin (Fig. B2). Statistical table of the effects of coating and storage time on banana quality, including browning index, respiration rate, weight loss, firmness, total soluble solids content, titratable acidity, ripening index, and pH (Tables B1 and B2) and further experimental details (Sections A1–A4). See DOI: https://doi.org/10.1039/d5ra09755j.
