# Supplementary material for: Pectin-rich purple passion fruit mesocarp/agarose: film properties and impact of coatings on banana preservation
Source: RSC Adv. 2026 Apr 23;16(23):21320–32. doi: 10.1039/d5ra09755j (PMC13103863; doi:10.1039/d5ra09755j)
Supplement: RA-016-D5RA09755J-s001 [file RA-016-D5RA09755J-s001.pdf]

Supplementary Information (SI) for RSC Advances.
This journal is © The Royal Society of Chemistry 2026

SUPPLEMENTARY FILE

Pectin-rich purple passion fruit mesocarp/agarose: Film properties and impact of coatings on banana preservation

Bao-Tran Pham-Tran[a,b], Nhu-Quynh Thi Nguyen[c], Nhu-Ngoc Quynh Nguyen[c], Long Giang Bach[a,b,*], Thuong Thi Nguyen[b,d,*]

[a] *Nguyen Tat Thanh University Center for Hi-Tech Development, Saigon Hi-Tech Park, Ho Chi Minh City, Vietnam*

[b] *Institute of Applied Technology and Sustainable Development, Nguyen Tat Thanh University, Ho Chi Minh City 71516, Vietnam*

[c] *Faculty of Chemical Engineering and Food Technology, Nong Lam University, Ho Chi Minh City 70000, Vietnam*

[d] *Faculty of Applied Science and Technology, Nguyen Tat Thanh University, Ho Chi Minh City 71514, Vietnam*

*Corresponding authors: blgiang@ntt.edu.vn (Long Giang Bach) and nthithuong@ntt.edu.vn (Thuong Thi Nguyen)

Section A.1. Pectin characterization.

The yield of pectin was calculated using the following formula:

$$Pectin\ yield\ (\%) = \frac{\text{Weight of dried pectin (g)}}{\text{Weight of dried passion fruit mesocarp powder (g)}} \times 100 \qquad (A.1)$$

The moisture content of the passion fruit mesocarp and pectin powder was determined by measuring the weight loss after drying the samples in an oven at 105 °C until a constant weight was achieved. The moisture content, expressed as a percentage, was calculated using the following Eq. (A.2):

$$Moisture\ content\ (\%) = \frac{W_{initital\ weight} - W_{weight\ after\ drying}}{W_{initial\ weight}} \times 100 \qquad (A.2)$$

Ash content of the passion fruit mesocarp and pectin powder was determined according to TCVN 5351:1991 standard. The porcelain crucible is washed and then heated in a furnace at 550 °C until constant mass, cooled in a desiccator, and weighed (W_1). 5 g of the material is put

into the porcelain crucible, weighed (W_2), and placed in a furnace at 550 °C. Heated until white ash, cooled in a desiccator, and weighed. Continue heating at the above temperature for 30 min, then cool in a desiccator and weigh until constant mass (W_3). Ash content is calculated by the formula:

$$Ash\ content\ (\%) = \frac{W_3 - W_1}{W_2 - W_1} \times 100 \qquad (A.3)$$

The antioxidant activity of the obtained pectin was determined by determining the scavenging ability using DPPH$^{\bullet}$ (2,2-diphenyl-1-picrylhydrazyl). Briefly, 0.2 g of the pectin powder was completely dissolved in 10 mL of distilled water, then 0.3 mM ethanolic DPPH$^{\bullet}$ solution was added with a volume ratio of 1:1 and shaken vigorously for 15 seconds before being left in the dark for 30 minutes. The absorbance value at 517 nm was measured using a UV-VIS Metash UV-510 (Shanghai Metash Instruments Co.). The DPPH$^{\bullet}$ radical scavenging ability was calculated using Eq. (A.4):

$$DPPH\ radical\ scavenging\ (\%) = \frac{Abs_{DPPH} - Abs_{sample}}{Abs_{DPPH}} \times 100 \qquad (A.4)$$

Section A.2. Film characterization

A.2.1. Thickness and color. The thickness was measured in 4 random locations (one in the center and three times at the edge of the as-prepared films) using a No.7313 thickness gauge (Mitutoyo Co.) to the nearest 15 μm.

Film color was assessed using the CR-400 colorimeter (Konica Minolta Holdings, Inc.) with a CIE LAB scale in the white standard plate (L = 93.59; a = −1.46; b = 4.69). Film color was expressed as L (lightness, ranged from 0 (black) to 100 (white)], a [greenness−redness, ranged from −60 (green) to +60 (red)], and b [blueness−yellowness, ranged from −60 (blue) to +60 (yellow)] values. The total color difference (ΔE), whiteness index (WI), and yellowness index (YI) of the composite films were determined and referred to the CH films using Eq. (A.5), Eq. (A.6), and Eq. (A.7).[1]

$$\Delta E = \sqrt{\left(L_{CH-sCeO_2} - L_{CH}\right)^2 + \left(a_{CH-sCeO_2} - a_{CH}\right)^2 + \left(b_{CH-sCeO_2} - b_{CH}\right)^2} \qquad (A.5)$$

$$WI = 100 - \sqrt{\left(100 - L_{CH-sCeO_2}\right)^2 + {a_{CH-sCeO_2}}^2 + {b_{CH-sCeO_2}}^2} \qquad (A.6)$$

$$YI = 142.86 \times \frac{b}{L} \qquad (A.7)$$

A.2.2. Fourier-transform infrared (FTIR) spectrometry and X-ray Diffraction (XRD). FTIR spectra of as-prepared films were measured using an Agilent Cary 630 spectrometer (Agilent Technologies Inc.) equipped with ATR. XRD of as-prepared films was obtained using D2 PHASER-XRD equipment (Bruker Co.) using Cu-Kα as a radiation source to determine their crystal structure.

A.2.3. Mechanical properties. Mechanical behavior of the as-prepared films (12 × 15 mm) was carried out by using a YM-H35 Universal material testing machine (Yang Yi Technology Co., Ltd.) with a static load cell of 5 kN, a cross-head speed of 10 mm/min, and a gauge length of 50 mm following the ASTM D882 standard.

A.2.4. Water barrier. Water contact angle measurements were performed using a CAM 101 surface tension meter (KSV Instruments Ltd.).

Water vapor permeability (WVP) was estimated using the method reported by Zhang et al. [2] with some minor modifications. A testing cup containing 100 mL of distilled water was sealed with various as-prepared films to obtain the initial mass. These cups were then stored at 25 °C in a desiccator holding 100 g of silica gel and were weighed every 24 h for 2 d. The WVP of as-prepared films was calculated using the formula below:

$$WVP\ (g\ s^{-1}\ m^{-1}\ Pa^{-1}) = \frac{\Delta w \times x}{t \times A \times \Delta P} \qquad (A.8)$$

where Δw is the reduced weight of the cup (g), x represents the thickness of the as-prepared film (m), t is the measurement time (s), A represents the contact area of the as-prepared film and the cup (m^2), and ΔP is the corresponding saturated vapor pressure at 25 °C.

The moisture content (MC) was evaluated using the methodology outlined in our prior work. [3] The 400 mm^2 square film specimens were weighed (W_1). After that, the film was dried for 24 h at 70 °C and weighed to obtain the ultimate dry mass (W_2). Equations were used to determine the moisture content.

$$MC\ (\%) = \frac{W_1 - W_2}{W_1} \times 100 \qquad (A.9)$$

A.2.5. Oxygen permeability. The films were used to seal the mouth of a glass bottle (2 cm in diameter) containing 3 g of deoxidizing agent (reduced iron powder, sodium chloride, and activated carbon). After that, the bottle was placed in a desiccator saturated with barium chloride solution (90% RH) for 48 h. The oxygen permeability (OP) was calculated using the following formula:[4]

$$OP\ (mol/m.s.Pa) = \frac{\frac{\Delta m}{M_{O2}} \times d}{t \times A \times \Delta P} \qquad (A.10)$$

where Δm is the added weight of the oxygen (g), d is the thickness of the films (m), t is the time (s), A is the area of the weighing bottle mouth (m^2), and ΔP is the corresponding saturated vapor pressure at 25 °C and 90%RH.

Section A.3. Characterization of Laba bananas during storage. *A.3.1. Sensory evaluation***.** Sensory evaluation of appearance quality, texture, taste, odor, and overall acceptability was conducted using a five-point hedonic scale with minor modifications.[5] The hedonic evaluation scale was ordered as follows: (1) dislike extremely, (2) dislike slightly, (3) neither like nor unlike, (4) like moderately, and (5) extremely like. The trained evaluators, including staff and undergraduate students, all of whom were under the age of 30, conducted each evaluation alone in separate rooms. All panelists evaluated all treatment groups (Control, PPMP-A0.5, PPMP-A1, and PPMP-A1.5) in a single session. The four sample presentation order was randomized and balanced among the evaluators to minimize potential bias.

A.3.2. Browning index. Peel color was estimated with the color recorder CR-400 (Konica Minolta Holdings, Inc.). The color values were determined using the CIE Lab space. The browning index (BI) of the banana during storage was calculated as follows:[6]

$$BI = \frac{a + 1.75L}{5.645L + a - 3.012b} \times \frac{0.31}{0.172} \times 100 \qquad (A.11)$$

A.3.3. CO_2 production. The banana respiration rate was measured based on the CO_2 production rate. The measurement was carried out individually with the GS6000 headspace analyzer (Illinois Instruments Inc.), after the bananas were kept for 2 h in the 2 L airtight container. The amount of CO_2 produced was expressed as mg/kg.h.

A.3.4. Weight loss. The weight of the bananas was recorded every 2 days during the storage period using the F6001B electronic laboratory balance with 0.01 accuracy (Huanghua Faithful Instrument Co., Ltd.), and the weight loss percentage of each group was calculated according to Eq. (A.12):

$$Weight\ loss\ (\%) = \frac{W_{day\ 0} - W_{sampling\ day}}{W_{day\ 0}} \times 100 \qquad (A.12)$$

A.3.5. Hardness. The banana pulp compression test was performed using the TA.XTplus texture analyzer (Stable Micro Systems Co., Ltd.). The tests were performed with the 6 mm diameter spherical stainless-steel probe, 10 mm penetration distance, and 5 mm s^{-1} speed. The

measurement was made on 5 bananas in the middle position to calculate the average hardness, and the displayed results were recorded as gf and converted into Newton (N).

A.3.6. Total soluble solids content, titratable acidity, ripening index, and pH. The total soluble solids content (TSS) of banana pulp was determined using the PAL-1 digital refractometer model (Atago Co., Ltd.), and the results were expressed in %.

The titratable acidity (TA) of banana juice was determined based on the earlier procedure.[7] In detail, a 10 g pulp banana sample was homogenized with 100 mL of distilled water for 60 s. The mixture was filtered with cotton wool to obtain the filtrate. After that, 10 mL of juice solution was titrated with 0.1 N NaOH (using 1% phenolphthalein as an indicator). The titration was stopped when the solution changed from colorless to pink without fading within 30 s, the final burette volume was recorded, and the TA value was calculated using the formula Eq. (A.13) with 0.067 as the milliequivalent factor of the malic acid in banana.

$$TA\ (\%\ malic\ acid) = \frac{V_{NaOH\ for\ titration} \times C_{N\ NaOH} \times 0.067}{m_{pulp} \times V_{juice\ for\ titration}} \times 100 \qquad (A.13)$$

The ripening index was calculated based on TSS/TA.

The pH of the above banana juice was measured using HI2211-02 bench pH meters (Hanna Instruments Inc.).

A.3.7. Microscopic characterization. Surface and cross-sectional morphology of the coating layer was determined using scanning electron microscopy (SEM) on JSM-IT200 equipment (JEOL Ltd.). Banana peels, after being coated on day 0, were freeze-dried by sublimation vacuum before testing.

Section A.4. Statistical analysis. Data analysis was performed using Statgraphics Centurion software, version 20 for Windows. Results were presented as mean ± standard deviation, and statistical significance was evaluated using the Tukey HSD test ($p < 0.05$). Different letters in the same column of tables or the same graph indicate statistically significant differences ($p < 0.05$) between samples.

References

1 C. Varela, F. Aghababaei, M. Cano-Sarabia, L. Turitich, A. J. Trujillo and V. Ferragut, *LWT*, 2022, **162**, 113493. DOI:10.1016/j.lwt.2022.113493.

2 W. Zhang, X. Li and W. Jiang, *Int. J. Biol. Macromol.*, 2020, **154**, 1205–1214. DOI:10.1016/j.ijbiomac.2019.10.275.

3 T. T. Nguyen, B.-T. T. Pham, H. N. Le, L. G. Bach and C. N. H. Thuc, *Food Packag. Shelf Life*, 2022, **32**, 100830. DOI:10.1016/j.fpsl.2022.100830.

4 J. Wang, D. J. Gardner, N. M. Stark, D. W. Bousfield, M. Tajvidi and Z. Cai, *ACS Sustain. Chem. Eng.*, 2018, **6**, 49–70. DOI:10.1021/acssuschemeng.7b03523.

5 M. Harich, B. Maherani, S. Salmieri and M. Lacroix, *Food Control*, 2018, **85**, 29–41. DOI:10.1016/j.foodcont.2017.09.018.

6 T. Wang, X. Zhai, X. Huang, Z. Li, X. Zhang, X. Zou and J. Shi, *Food Packag. Shelf Life*, 2023, **39**, 101133. DOI:10.1016/j.fpsl.2023.101133.

7 R. Thakur, P. Pristijono, M. Bowyer, S. P. Singh, C. J. Scarlett, C. E. Stathopoulos and Q. V. Vuong, *Lwt*, 2019, **100**, 341–347. DOI: 10.1016/j.lwt.2018.10.055.

Section B.1. Results

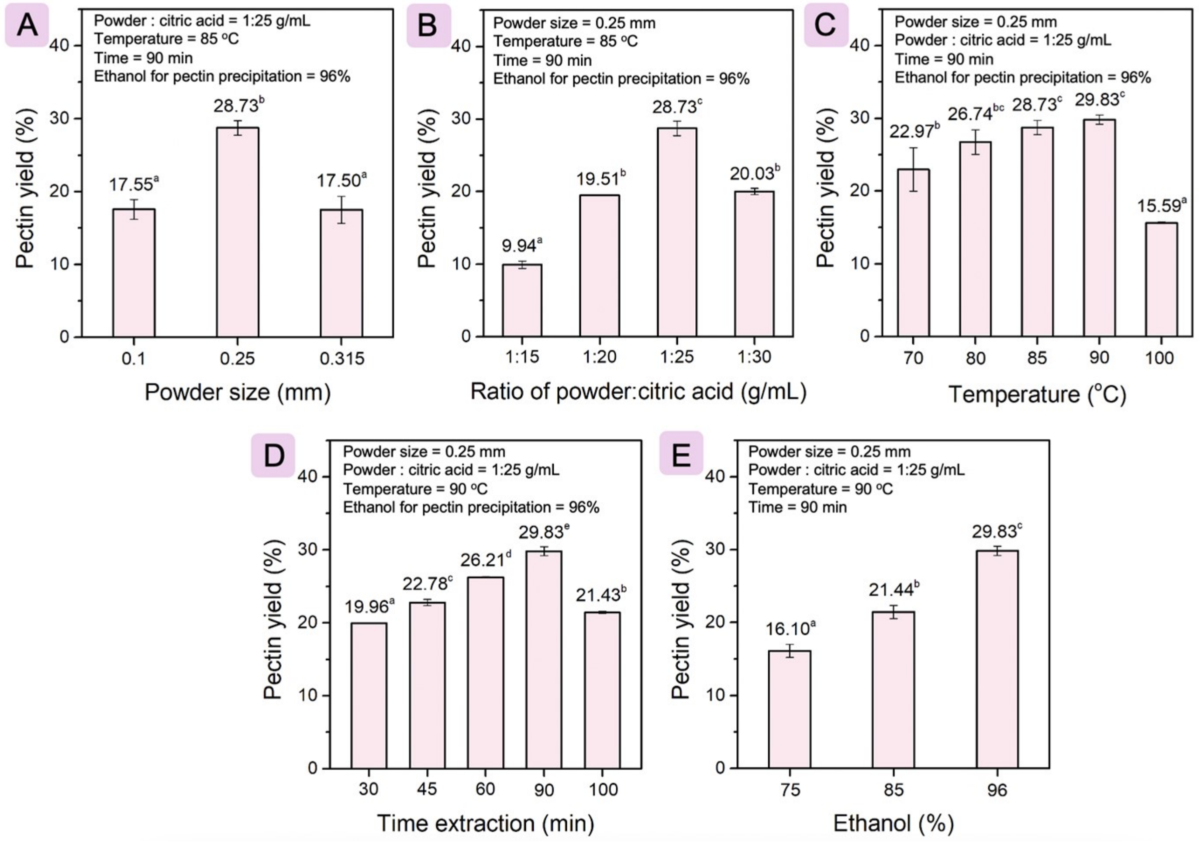

Figure B1. The influence of dry powder size (A), ratio of power:citric acid (B), temperature extraction (C), time extraction (D), and ethanol concentration used for pectin precipitation (E) on the pectin extraction capacity.

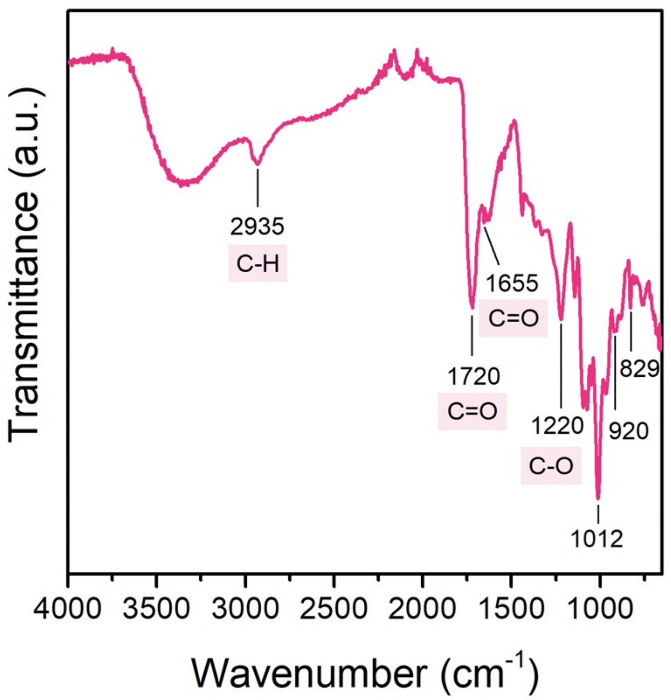

Figure B2. FTIR of extracted pectin.

Table B1. Effects of PA coatings on browning index, respiration rate, weight loss, and firmness of banana during storage at 25 °C and 64% RH.

Coating × day	Browning index	CO_2 production (mg/kg.h)	Weight loss (%)	Firmness (N)
Control × day 0	49.30±1.87 *ab*	325.84±13.28 *a*	0.00±0.00 *a*	44.13±0.10 *a*
PPMP-A0.5 × day 0	46.10±2.53 *a*	325.84±13.28 *a*	0.00±0.00 *a*	44.13±0.10 *a*
PPMP-A1 × day 0	44.90±2.21 *a*	325.84±13.28 *a*	0.00±0.00 *a*	44.13±0.10 *a*
PPMP-A1.5 × day 0	47.24±2.99 *ab*	325.84±13.28 *a*	0.00±0.00 *a*	44.13±0.10 *a*
Control × day 2	66.21±3.03 *cde*	432.06±6.13 *ef*	6.59±0.21 *c*	23.96±1.65 *ef*
PPMP-A0.5 × day 2	71.56±5.45 *cdefg*	395.97±15.14 *bcdef*	4.61±0.27 *b*	28.62±0.21 *cd*
PPMP-A1 × day 2	64.79±3.80 *cd*	383.84±7.71 *bcde*	4.75±0.12 *b*	30.83±0.93 *c*
PPMP-A1.5 × day 2	60.42±3.40 *bc*	365.34±4.30 *abc*	4.14±0.00 *b*	38.04±2.90 *b*
Control × day 4	85.70±6.68 *hi*	411.70±8.59 *cdef*	12.43±0.26 *e*	16.11±0.34 *h*
PPMP-A0.5 × day 4	78.16±4.56 *efgh*	440.17±0.07 *f*	9.69±0.26 *d*	23.19±0.10 *efg*
PPMP-A1 × day 4	71.18±1.12 *cdefg*	420.72±2.02 *def*	9.67±0.42 *d*	25.99±0.10 *de*
PPMP-A1.5 × day 4	67.03±4.00 *cdef*	373.37±28.50 *abcd*	7.79±0.22 *c*	31.72±0.74 *c*
Control × day 6	95.46±3.33 *i*	357.75±7.90 *ab*	18.04±0.25 *gh*	12.26±0.12 *i*
PPMP-A0.5 × day 6	83.30±3.75 *ghi*	380.75±6.35 *bcd*	14.90±0.33 *f*	16.19±0.19 *h*
PPMP-A1 × day 6	78.03±1.59 *efgh*	407.00±2.80 *bcdef*	14.00±0.86 *f*	20.08±0.36 *g*
PPMP-A1.5 × day 6	67.31±2.86 *cdef*	419.97±22.40 *def*	11.69±0.44 *e*	21.96±0.68 *fg*
Control × day 8	-	-	-	-
PPMP-A0.5 × day 8	93.96±4.08 *i*	363.53±23.54 *abc*	19.14±0.37 *h*	8.67±0.35 *j*
PPMP-A1 × day 8	79.89±1.59 *fgh*	399.23±4.39 *bcdef*	17.48±1.04 *g*	10.46±0.23 *ij*
PPMP-A1.5 × day 8	75.20±2.64 *defgh*	359.07±12.36 *ab*	14.67±0.56 *f*	16.22±0.48 *h*
Significant differences				
Coating (C)	***	ns	***	***
Storage time (D)	***	***	***	***
C × D	ns	ns	ns	ns

*** Mean values are obtained from triplicate samples. Different letters within each column indicate a significant difference ($p < 0.05$) between coating formulation and storage using the Tukey HSD test.

ns: non-significant.

Table B2. Effects of PA coatings on total soluble solids content (TSS), titratable acidity (TA), ripening index, and pH of banana during storage at 25 °C and 64% RH.

Coating × day	TSS (%)	TA (% malic acid)	Ripening index	pH
Control × day 0	17.00±0.22 *a*	0.491±0.016 *a*	34.62±0.70 *a*	4.70±0.00 *a*
PPMP-A0.5 × day 0	17.00±0.22 *a*	0.491±0.016 *a*	34.62±0.70 *a*	4.70±0.00 *a*
PPMP-A1 × day 0	17.00±0.22 *a*	0.491±0.016 *a*	34.62±0.70 *a*	4.70±0.00 *a*
PPMP-A1.5 × day 0	17.00±0.22 *a*	0.491±0.016 *a*	34.62±0.70 *a*	4.70±0.00 *a*
Control × day 2	21.80±0.20 *cde*	0.369±0.027 *defg*	59.08±4.29 *fg*	4.96±0.04 *b*
PPMP-A0.5 × day 2	18.30±0.28 *ab*	0.413±0.016 *bcde*	44.31±1.45 *bcd*	4.78±0.03 *a*
PPMP-A1 × day 2	17.63±0.90 *a*	0.447±0.016 *abc*	39.44±2.94 *abc*	4.73±0.03 *a*
PPMP-A1.5 × day 2	17.10±0.00 *a*	0.469±0.000 *ab*	36.46±0.01 *ab*	4.71±0.01 *a*
Control × day 4	22.77±0.80 *def*	0.357±0.016 *efg*	63.78±2.57 *gh*	5.08±0.01 *d*
PPMP-A0.5 × day 4	20.60±0.57 *c*	0.391±0.016 *cdef*	52.68±3.18 *def*	4.98±0.03 *bc*
PPMP-A1 × day 4	18.53±0.09 *ab*	0.424±0.016 *bcd*	43.70±1.44 *bc*	4.96±0.01 *b*
PPMP-A1.5 × day 4	17.90±0.00 *a*	0.447±0.016 *abc*	40.04±1.38 *abc*	4.93±0.01 *b*
Control × day 6	23.13±1.62 *ef*	0.335±0.000 *fgh*	69.04±4.84 *h*	5.30±0.02 *fg*
PPMP-A0.5 × day 6	21.63±0.47 *cde*	0.357±0.016 *efg*	60.59±1.28 *fg*	5.14±0.01 *de*
PPMP-A1 × day 6	20.17±0.33 *bc*	0.380±0.016 *def*	53.08±0.89 *efg*	5.10±0.02 *d*
PPMP-A1.5 × day 6	19.97±0.31 *bc*	0.369±0.016 *defg*	48.35±1.08 *cde*	5.06±0.03 *cd*
Control × day 8	-	-	-	-
PPMP-A0.5 × day 8	24.10±0.00 *f*	0.290±0.016 *h*	83.10±5.53 *i*	5.37±0.05 *g*
PPMP-A1 × day 8	21.53±0.09 *cde*	0.313±0.016 *gh*	68.78±0.31 *hi*	5.30±0.02 *fg*
PPMP-A1.5 × day 8	20.93±0.25 *cd*	0.335±0.027 *fgh*	62.48±5.62 *gh*	5.21±0.02 *ef*
Significant differences				
Coating (C)	***	***	***	***
Storage time (D)	***	***	***	***
C × D	ns	ns	ns	ns

*** Mean values are obtained from triplicate samples. Different letters within each column indicate a significant difference ($p < 0.05$) between coating formulation and storage time using the Tukey HSD test.

ns: non-significant.
